# Single‐fraction image‐guided robotic radiosurgery efficiently controls local prostate cancer recurrence after radical prostatectomy

**DOI:** 10.1002/bco2.32

**Published:** 2020-08-05

**Authors:** A. Spek, A. Graser, G. Habl, A. Muacevic, C. Fuerweger, M. Seitz, A. Haidenberger

**Affiliations:** ^1^ Department of Urology Ludwig Maximilian University Munich Germany; ^2^ Radiologie Muenchen Munich Germany; ^3^ Department of Radiation Therapy Technical University of Munich Munich Germany; ^4^ European Cyberknife Center Munich Munich Germany; ^5^ Uroclinic Bogenhausen Munich Germany

**Keywords:** prostate cancer, prostatectomy, PSA, recurrence, SABR

## Abstract

**Purpose:**

To assess the therapeutic potential of single‐fraction robotic stereotactic ablative body radiotherapy (SABR) in patients with locally recurrent prostate cancer (PC) after radical prostatectomy (RP).

**Materials and methods:**

We included 35 patients with biochemical failure after RP with single‐site local recurrence in the prostate bed diagnosed by PSMA PET/CT. About 20/35 pts had previously received post‐surgical adjuvant radiation therapy.

High‐resolution multiparametric magnetic resonance imaging (mpMRI) for exact visualization of tumor tissue was performed at 1.5 (n = 23; Siemens Magnetom Aera) or 3 Tesla (n = 12; Siemens Magnetom VIDA, Siemens Healthineers, Erlangen, Germany). Using the MRI and PET/CT dataset for planning, SABR was carried out after ultrasound‐guided placement of a single gold fiducial marker at the site of tumor recurrence using a CyberKnife M6 unit (Accuray Inc., Sunnyvale, USA). Due to the high diagnostic accuracy of PSMA PET/CT and mpMRI, pre‐SABR biopsy of tumor tissue was not deemed necessary. PSMA PET/CT performed in median 88 days before SABR confirmed the absence of distant metastases. MpMRI was performed at a median of 22 days prior to the intervention. SABR was performed in a single fraction with a dose of 20 (5/35), 21 (27/35) or 22 (3/35) Gy. Follow‐up serum PSA was measured every 3 months thereafter.

**Results:**

Median patient age was 72 years (57‐80 years) and median time from RP to SABR was 96.8 months (IQR, 69.3‐160.2). Median serum PSA before SABR was 1.38 ng/mL (IQR 0.75‐2.72). At 3 months, median PSA had dropped significantly in 27/35 patients to a median of 0.35 ng/mL (IQR 0.25‐0.68). At 6 months, 30/35 patients showed biochemical response to SABR, while five patients were progressing: three had systemic disease on PSMA PET/CT, while two patients had rising PSA values without a visible correlate on PET/CT. The median follow‐up time was 16 months. Grade 1 genitourinary (GU) toxicity was reported in 3/35 patients (9%) and grade 1 gastrointestinal (GI) toxicity in 2/35 patients (6%), respectively.

**Conclusion:**

SABR is an efficient new treatment option in the management of single‐site local recurrent PC without the evidence of systemic disease; due to its very low toxicity, it is an alternative to surgical re‐treatment or other focal therapies. It can significantly delay the onset of androgen deprivation therapy (ADT) in biochemical failure after radical prostatectomy.

## 1 INTRODUCTION

Radical prostatectomy (RP) is a frequently performed standard‐of‐care treatment option in the management of patients with prostate cancer, which is the most common malignancy in males throughout the Western hemisphere; its incidence in Germany is more than 57 000 cases per year.^1^ After curative therapy, patients are routinely monitored by repetitive serum PSA level measurements. If the biochemical recurrence of prostate cancer is documented on at least two consecutive PSA measurements, further investigations are needed to clarify if there is a local recurrence or systemic disease with distant metastases. Approximately 17‐30% of patients develop a detectable serum PSA 5 years after RP; the actual recurrence rate rises with increasing tumor size, resection status, and corresponds to tumor Gleason score at RP.^2^, ^3^, ^4^ In men who did not receive adjuvant radiation therapy, salvage radiotherapy is considered the treatment of choice.^5^ In patients who already underwent adjuvant radiation therapy or in whom systemic recurrence is suspected, androgen deprivation therapy (ADT) is a commonly used therapeutic option.

Recently, there is growing interest in early local therapy of recurrence in the prostate bed which can be diagnosed at high sensitivity since PSMA PET/CT has been introduced into a clinical routine.^6^ Any type of image‐guided local ablative treatment delays the onset of ADT and other systemic therapies in the majority of patients; also, local therapy of PCA recurrence might prolong overall survival and improve prognosis.^7^ Local therapies like radiation therapy or surgery differ from a systemic approach in terms of side effects and efficacy. If local recurrence in the prostate bed is diagnosed on clinical examination, ultrasound, or imaging, salvage radiotherapy or surgical excision can be considered as locally ablative treatment options. In most cases, surgical removal of local PC recurrence is technically challenging and associated with significant morbidity.^8^, ^9^ Anatomical landmarks are altered in postsurgical patients and precise localization and excision of tumor tissue may be impossible. Standard external beam radiation therapy (EBRT) for local recurrence of prostate cancer after RP is well tolerated but the whole prostate bed is irradiated, potentially leading to higher grades of side effects due to higher doses to the affected organs at risk (OAR).

Stereotactic ablative body radiation (SABR), moreover, is a novel technique to treat small lesions in a single fraction with high ablative doses and very low toxicity^10^; its patient acceptance is high as the necessary ablative radiation dose can mostly be applied in one single fraction of 20 to 22 Gy.^11^, ^12^, ^13^ We sought to assess the efficacy of this new treatment option in patients with single‐site local recurrence of PC after RP +/− adjuvant RT.

## 2 MATERIALS AND METHODS

Between April 2016 and June 2019, 35 patients with isolated local recurrence after RP and initial adjuvant radiation therapy (n = 20/35) were included in the present study. Inclusion criteria were defined as follows: (a) biochemical recurrence after primary treatment of PCA with radical prostatectomy according to the guidelines; (b) written informed consent; (c) absence of distant metastases; (d) single‐site local recurrence on PSMA PET/CT and high‐resolution multiparametric MRI (mpMRI). IRB approval was waived.

Before treatment with stereotactic radiosurgery, a high‐resolution multiparametric MRI examination for the exact visualization of tumor tissue was performed at a field strength of 1.5 Tesla (Siemens Magnetom Aera 48‐channel/2016, n = 23) or 3.0 Tesla (Siemens Magnetom Vida 64‐channel / 2018, Siemens Healthineers, Erlangen, Germany; n = 12) at a median of 22 days prior to the intervention. All patients underwent PSMA PET/CT at a median of 88 days before therapy to confirm the absence of distant metastases and to localize PC recurrence. An additional biopsy of tumor tissue diagnosed on PSMA PET/CT was not performed as MRI confirmed local recurrence at the same anatomical location in all 35 patients. Without this mpMRI correlation, SABR cannot be safely performed as the target volume cannot be defined with a sufficient spatial resolution on PSMA PET/CT.

**Figure 1:**
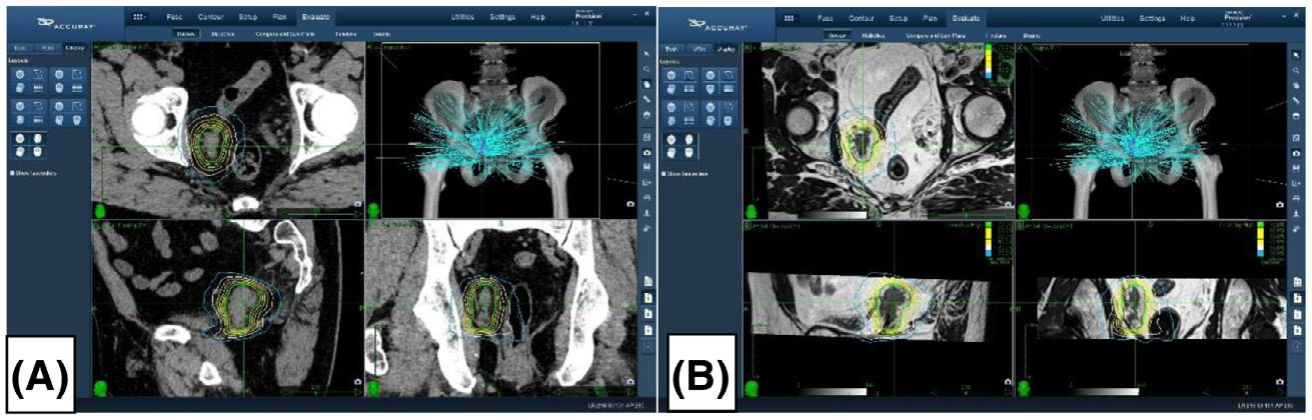
Treatment plan based on the planning CT (A), PET/CT and MRI (B) dataset

**Figure 2:**
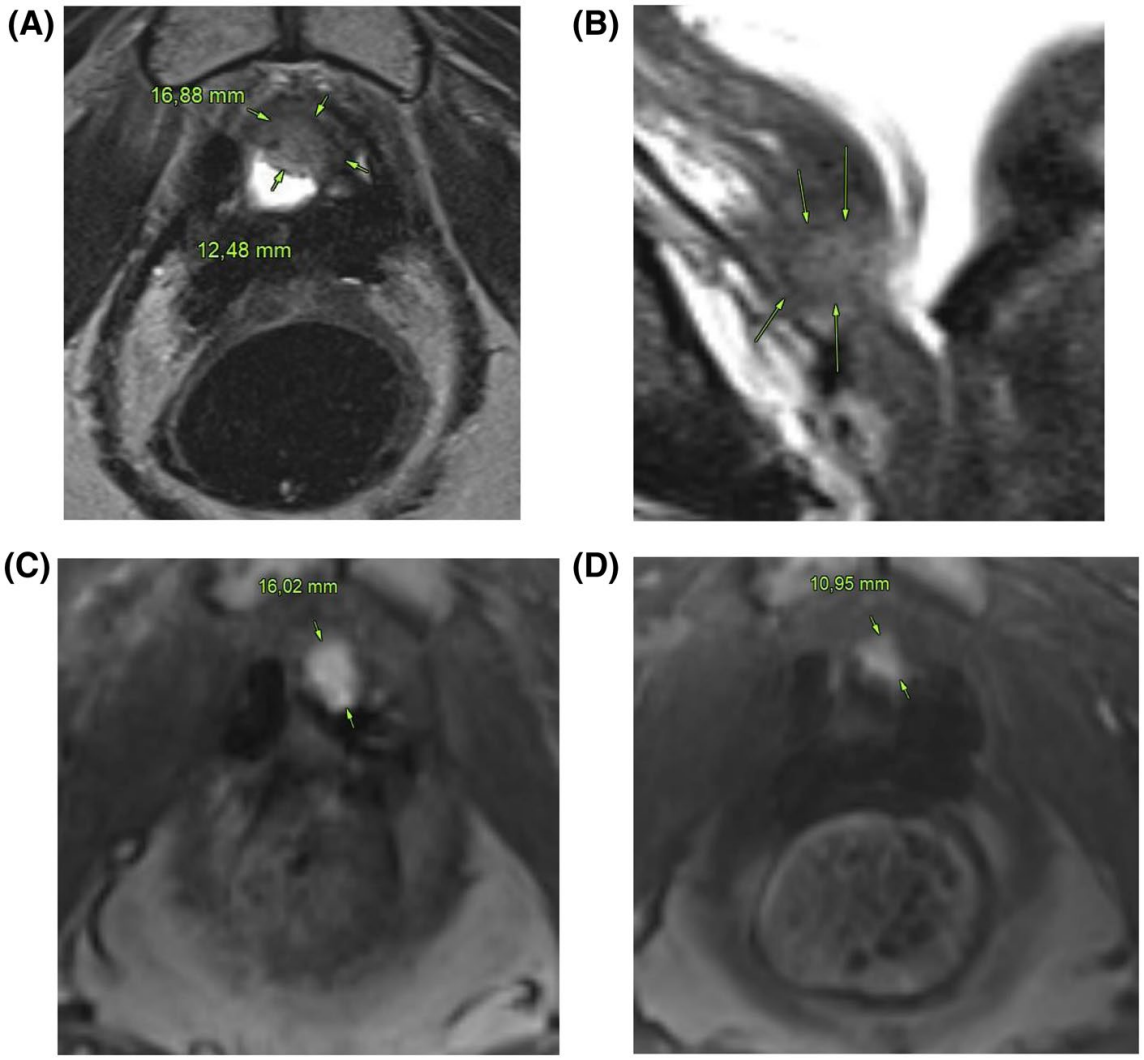
High‐resolution 3D SPACE 3 Tesla MR image shows a 17 × 12 mm soft tissue mass at the ventral aspect of the anastomosis, consistent with local tumor recurrence after radical prostatectomy (A). A sagittal T2w image demonstrates the anatomic relationship of the lesion to the bladder wall and the urogenital diaphragm (B). Pre SABR (C), the lesion is markedly hypervascular on a single transaxial arterial phase MR perfusion image measuring 16 mm in its greatest diameter; 6 months after treatment (D), vascularity of the lesion is significantly reduced and the size of the enhancing portion of the mass has shrunk to 11 mm consistent with response to the treatment

An individualized treatment plan was developed for all patients based on the planning CT, PET/CT and MRI dataset, compare Figure 1. After high‐resolution ultrasound‐guided placement of two gold fiducial markers, a non‐contrast‐enhanced computed tomography scan was performed prior to SABR treatment. MRI and PET/CT data were fused to this basic planning CT for optimal anatomical tumor delineation (Siemens AS 20, Siemens Healthineers, Forchheim, Germany). Tumor target definition was carried out using the Precision 2.0 planning software (Accuray Inc., Sunnyvale, CA, USA) included macroscopically visible tumor tissue on MRI/PET/CT and a 2‐3 mm safety margin in all directions, see Figure 2.

SABR was carried out using a CyberKnife M6 unit (Accuray Inc., Sunnyvale, USA). Radiosurgical treatment was performed in a single fraction with a median dose of 21 Gy (range, 20‐22 Gy).

Treatment response and follow‐up were assessed by initial serum PSA measurement 3 months after SABR, and every 3 months thereafter. In cases with rising PSA levels a repeated PSMA PET/CT was performed. Side effects of SABR were recorded at patient visits every 3 months after SABR using a standardized patient questionnaire.

## 3 RESULTS

Median patient age at intervention was 72 years (range, 57‐80 years). After radical prostatectomy, 20 high‐risk patients had undergone adjuvant radiation therapy due to an unfavorable initial clinical tumor stage of pT3a and over and/or positive surgical resection margins. Adjuvant RT was performed in median 23 months (IQR 7‐42 months) after RP with doses between 64‐70 Gy. The median time between RP and SABR was 96.8 months (IQR, 69.3‐160.2 months). Patient characteristics at the time of primary treatment are summarized in Table 1. The median serum PSA level before SABR was 1.38 ng/mL (IQR 0.75–2.72). None of the men was receiving ADT at the time of biochemical recurrence. SABR was performed in a single fraction with a dose of 20 (5/35), 21 (27/35), or 22 (3/35) Gy. The first clinical follow‐up of patients was performed 3 months after SABR including the serum PSA measurement. Three months after SABR, the median PSA level had dropped significantly in 27/35 patients from a median of 1.47 ng/mL (IQR 0.82‐2.78 ng/mL) to a median of 0.35 ng/mL (IQR 0.25‐0.68 ng/mL); at this early timepoint, three patients showed a transient increase in PSA values (patient 1: 0.75 to 0.96 ng/mL; patient 2: 0.79 to 1.45 ng/mL; patient 3: 0.62 to 0.71 ng/mL), while at 6 months, these patients had decreasing PSA values (patient 1: 0.11 ng/mL; patient 2: 0.3 ng/mL; patient 3: 0.25 ng/mL). In summary, at 6 months, 30/35 patients showed biochemical response to SABR treatment, while five patients were progressing; three had systemic disease on PSMA PET/CT, while the treated tumor did not show PSMA uptake, and two patients developed rising PSA values without a visible correlate on PET/CT imaging.

**Table 1:** Characteristics at primary treatment (rPx)

	n	%
*Age at initial diagnosis*		
≤60	20	57
>60	15	43
*Primary treatment*		
rPx	15	57
rPx + RT	20	43
*pT*		
pT2a	2	6
pT2b	6	17
pT2c	7	20
pT3a	13	37
pT3b	7	20
*Gleason*		
6	4	11
7	22	63
8	4	11
9	5	14

IPSA levels before surgery, PSA levels before SABR, and PSA nadir after SABR for each patient as well as tumor characteristics at initial treatment are shown in Table 2.

**Table 2:** PSA values and characteristics at first treatment

No	Age at first diagnosis	PSA before surgery	Gleason‐score	Stage	Adjuvant RT	PSA before SRS	SRS dose	PSA nadir
1	48	120	4 + 5	T3b	x	1.22	22	0.78
2	58	6.5	3 + 4	T2b		0.75	22	<0.03
3	74	15	4 + 5	T3b	x	1.7	21	1.57
4	66	31.34	3 + 3	T2c	x	4.01	21	1.45
5	57	8.2	4 + 3	T2a		1.36	21	0.03
6	64	6.11	3 + 4	T2c	x	1.38	21	0.07
7	57	9.2	3 + 4	T3a		10.71	20	0.33
8	62	11.5	3 + 4	T2c	x	0.9	22	0.07
9	61	5.4	3 + 3	T2a	x	2.75	21	0.79
10	61	8.27	3 + 4	T3a	x	0.75	21	0.13
11	53	18.2	5 + 4	T2c	x	2.8	21	0.31
12	54	9	4 + 3	T3a	x	1.47	20	0.16
13	57	63	4 + 5	T3b	x	0.68	21	0.87
14	62	16.4	3 + 4	T3a		5.47	20	12.8
15	51	13	3 + 4	T3a	x	2.8	21	0.1
16	51	8.82	3 + 4	T3b	x	0.6	21	7.00
17	46	5.8	4 + 3	T2b	x	17.28	21	1.49
18	60	13.5	3 + 4	T2b		0.74	21	0.02
19	54	8.9	4 + 4	T2b		2.68	21	0.12
20	76	5.82	4 + 3	T3a		0.79	21	0.303
21	63	4.3	3 + 3	T2b		1.57	21	0.55
22	68	6.15	3 + 4	T3a		1.62	21	5.42
23	59	6.8	3 + 4	T2c		0.5	21	0.33
24	62	5.1	4 + 3	T2c		0.48	21	0.09
25	52	11.7	4 + 4	T3b	x	1.87	21	0.13
26	67	7.86	4 + 5	T3a		7.86	21	0.65
27	74	13.9	4 + 3	T3a		0.62	21	0.245
28	50	18	4 + 4	T3b	x	1.16	21	0.378
29	55	6.62	3 + 4	T3a	x	0.88	21	0.5
30	75	7	3 + 4	T2c	x	0.11	21	0.07
31	59	15.3	3 + 3	T2b		3.19	21	0.67
32	61	30	3 + 4	T3b		2	20	0.34
33	56	6.3	4 + 3	T3a	x	0.3	21	0.34
34	57	9.5	4 + 3	T3a	x	0.51	21	0.001
35	60	14.45	4 + 4	T3a	x	1.45	20	1.35

The median follow‐up time was 16 months (6‐47 months). At the timepoint of censoring of data, all patients were still alive.

Toxicity following SABR was reported at every standard visit interval. Grade 1 genitourinary (GU) toxicity was reported in three patients (9%), thereof one patient developed acute grade 1 GU toxicity in terms of cystitis and two patients late toxicity manifesting as pollakisuria. G1 gastrointestinal (GI) toxicity occurred in two patients (6%). Both reported temporary and intermittent diarrhea at first control interval after SABR. Grade 2 or higher GI toxicity was not reported.

## 4 DISCUSSION

Biochemical recurrence after radical prostatectomy with or without adjuvant radiation therapy occurs commonly in daily clinical practice. It is well documented that 30‐35% of men develop a relapse within 10 years after the initial definitive treatment.^2^, ^14^, ^15^, ^16^ Guidelines recommend salvage RT with a total dose of at least 66 Gy of the prostate bed and pelvic lymph nodes as a first‐line treatment option if local recurrence is suspected^17^; mostly, this treatment option will be employed without prior imaging and its success is solely monitored by the measurements of the PSA value.^5^, ^18^, ^19^

If routine follow‐up PSA measurements demonstrate BCR of prostate cancer, the focus of recurrence can be exactly localized by means of PSMA PET/CT and mpMRI.^20^, ^21^ PSMA PET/CT allows the exclusion of distant metastases and detection of local recurrence at high sensitivities.^20^, ^21^ Also, PSMA PET/CT provides very high specificity, making histopathological confirmation unnecessary in the majority of cases.^22^, ^23^ This information can be exploited in order to tailor individualized treatment concepts including the ablation of local recurrent PC tissue or single metastases.^10^, ^24^

Conventional fractionated RT requires an average of 33‐35 single sessions in order to deliver the recommended minimum total dose of 66‐70 Gy; hypofractionated treatment schemes are currently under investigation. SABR is a relatively new option of a high conformal technique that allows to deliver a very high dose of extremely focused ionizing radiation to a small defined tissue target volume with lower risk of inducing collateral damage and toxicity to the surrounding tissues.^25^, ^26^ Single fraction body RT as delivered by Cyberknife technology involves one therapeutic session only, thereby improving patients' quality of life and compliance. SABR is a preferred technique to treat local recurrence in the prostate bed because motion artifacts in this area are frequent and the rectum is very close to the radiation field. Dynamic MRI sequences show significant peristalsis of the rectum and the prostate which may lead to inadvertent irradiation of the anterior rectal wall causing local toxicity. Image‐guided robotic radiosurgery is ideally suited as it compensates for the movement of the recurrent tumor. Conventional external radiation therapy cannot correct intra‐fraction tumor movement and therefore multiple fractions are needed for a safe application of relevant tumoricidal doses.^27^ Another therapeutic possibility is the surgical resection of the recurrent lesion: However, surgery after primary PC resection or radiotherapy is very difficult, technically challenging, and associated with significant morbidity. Currently, there are some studies describing a new technique for surgical resection of recurrent PC lesions by means of PSMA‐radioguided surgery.^9^, ^28^ This new technique helps to localize any metastatic lesion or local recurrence of PC during salvage surgery. It requires an intravenous injection of radioactive molecules before surgery and a gamma probe for intraoperative radioguidance. Radioguided surgery is performed by a few experts in specialized centers only and is associated with substantial operation times and postoperative regeneration periods. SABR for local PC recurrence, moreover, is characterized by very low toxicity and absence of surgical trauma.

Follow‐up after SABR is commonly performed with serum PSA level measurements in regular intervals every 3 months. Serum PSA is a very reliable and sensitive marker for detecting tumor tissue. It is well known that the response to RT leads to a delayed decrease in serum PSA. PSA nadir after primary radiotherapy with curative intent is reached 12‐18 months after therapy. We measured serum PSA every 3 months but in our trial in some cases post‐treatment PSA nadir was reached only after 6‐9 months. Therefore, it should be discussed when to control serum PSA after SABR to optimize treatment response monitoring.

In our study, none of the patients received ADT. Besides preserving life quality and prolonging the time to systemic therapy, increasing survival data is the desirable goal to achieve. RTOG 9601 showed improved OS when combining ADT to salvage RT in patients with PSA levels ≥0.7 ng/mL and/or additional risk factors such as Gleason Score ≥8.^29^ Combination of SABR and ADT should be considered in this constellation; the timing of ADT to RT has to be evaluated.

Our study has limitations due to the relatively small patient cohort and limited median follow‐up time of only 12 months; further observation of this patient population is needed to report long‐time outcomes and side effects. Nevertheless, we believe that we are able to show the clinical feasibility of this cutting‐edge therapeutic approach in selected PC patients with local recurrence only thereby prolonging the time to the onset of ADT.

## 5 CONCLUSION

Single‐fraction image‐guided SABR for recurrent PC after radical prostatectomy is a reliable and safe treatment option in patients with isolated local recurrence in the prostate bed. After the PSMA PET/CT‐based localization of tumor tissue, high‐resolution MRI allows for exact treatment planning. SABR offers excellent local tumor control and is very well tolerated based on its low toxicity profile.

## CONFLICTS OF INTEREST

The authors declare that they have no conflict of interest.

## ETHICS APPROVAL

IRB approval was waived.
